# Exploring the effects of perceived social support and psychological distress through mediation and multigroup analyses in work-related quality of life

**DOI:** 10.1038/s41598-024-81548-y

**Published:** 2025-01-03

**Authors:** Sıdıka Ece Yılmaz, Şenel Çıtak

**Affiliations:** 1 Rectorship/Career Planning Application and Research Center, Adana Alparslan Türkeş Science and Technology University, Adana, Turkey; 2 Faculty of Education/Department of Guidance and Psychological Counseling, Ordu University, Ordu, Turkey

**Keywords:** Work-related quality of life, Perceived social support, Psychological distress, Multigroup analysis, Mediation analysis, Gender differences, Psychology, Human behaviour

## Abstract

Work-related quality of life has emerged as a central focus in the field of occupational health due to its impact on individual well-being and overall quality of life. So, it is crucial to enhance the current theory by conducting a study on the concept across different sectors. Also, the specific mechanisms through which perceived social support influences work-related quality of life remain underexplored. The role of psychological distress as a potential mediator in this relationship has not been investigated. Thus, the study aimed to explore the mediating role of psychological distress in this relationship, while also examining potential gender differences. The study, conducted with 401 teachers through a questionnaire, utilized the AMOS program for data analysis. The findings supported the direct effects among the variables and the mediating role of psychological distress. Additionally, the impact of psychological distress on work-related quality of life was found to be higher for females, but no differences for other paths. The study contributes to the literature by addressing the gap in understanding the mechanisms regarding work-related quality of life. The results highlight the importance of considering psychological distress and gender differences in interventions aimed at improving work-related quality of life.

## Introduction

Work-related quality of life (WRQoL) has become a prominent focus in occupational and health psychology in recent years. This is due to its significant implications for employee well-being, job satisfaction, organizational success, and overall societal health^1–3^. Additionally, an individual’s work activities and experiences have a substantial effect on their work and social environment, as well as their physical and psychological well-being, at various stages of life. Employees frequently encounter challenges in separating between their work life and social life^4^. This challenge is affected by factors including stress, work-life balance, and job satisfaction, each of which significantly influences WRQoL^3,5^. Therefore, WRQoL can be affected by various factors. Accordingly, it is recommended that a thorough investigation be conducted, with a specific emphasis on different factors^6^.

The dependence between social life and work is unavoidable. For instance, a general sense of unease, characterized by psychological distress such as a lack of interest, and despair, is likely to have an impact on an employee’s work life. Given the potential for distraction and cognitive errors experiencing psychological distress, it is unavoidable that the quality of work processes may decline. Likewise, psychological distress presents a threat to all organizations. This means that examining psychological distress within the framework of WRQoL is believed to enhance the literature and have practical implications. The presence of perceived social support has a crucial role in dealing with psychological distress^7,8^. It has been found that social support can enhance an employee’s ability to cope with stressful conditions^9^. It is important to acknowledge that social support extends beyond colleagues and supervisors. There is a must to enhance the comprehension of how social support beyond the workplace influences individuals’ work experiences, thus necessitating an expansion of the research scope on social support^10^. For instance, social support derived from family, friends, and significant others significantly influences life satisfaction^11^. That means the implications of the concept should also be examined within the work environment. This study seeks to enhance the literature by examining the impact of non-work social support on WRQoL.

Upon examining the literature, researchers have explored the direct relationship between perceived social support and work-life balance^12^. A lack of research has been found in the literature that explicitly addresses its impact on WRQoL. This indicates that the impact of social support has been examined within a restricted framework, prompting further research on its influence on WRQoL. Individuals with insufficient social support in the workplace may experience diminished WRQoL, and a scarcity of resources may induce stress^13^. The study examines the correlation between perceived social support and WRQoL, and for the first time, it explores the role of psychological distress as a significant factor in this relationship. Furthermore, existing research indicates that females may encounter and react to stressors associated with work and distinctly receive social support from males, owing to a range of sociocultural, biological, and organizational factors^14^. Accordingly, it is essential to examine gender disparities in the association of perceived social support, psychological distress, and WRQoL.

Understanding the mediating role of psychological distress in the relationship between perceived social support and WRQoL and gender differences becomes crucial to implementing effective interventions in the workplace. Organizations can diversify their social support strategies and customize their assistance programs accordingly. For instance, non-work social assistance can be enhanced by initiatives such as family support programs, flexible working hours, or work-life balance regulations. Initiatives that enhance employees’ connections with family, friends, and significant others. Furthermore, interventions might be developed to address the impact of psychological distress on WRQoL based on gender. Consequently, the objective of this study is to examine the mediating role of psychological distress impacts the relationship between perceived social support and their WRQoL, and figure out if these impacts vary based on gender.

## Literature review

The WRQoL has gained significant importance in contemporary society. The immediate influence of work on individuals’ quality of life and general satisfaction underscores the significance of the work environment’s contribution to various aspects of life. Employees’ work experiences are decisive in areas such as general life satisfaction, psychological resilience, and social relations^15^. The spillover theory proposed by Leiter and Durup posits that an individual’s work experiences have the potential to influence other aspects of their life, particularly their social and personal lives, either beneficially or unfavorably^4^. Also, personal life experiences can impact work experiences. This theory establishes a crucial basis for comprehending the impact of daily life on WRQoL. Factors including time allocation in daily activities, stress levels, and interpersonal dynamics can influence an individual’s workplace motivation, job satisfaction social connections, and overall life satisfaction. Therefore, it is crucial to examine and comprehend the WRQoL to enhance individuals’ work experiences, relationships, and overall satisfaction with life. WRQoL refers to the level of satisfaction and sense of ease and security that employees experience in their professions. Aspects such as WRQoL, workplace physical conditions, wage, chances for promotion, job satisfaction, and workplace social life are all important factors to consider in the workplace. The structure is multidimensional due to its association with concepts^6^. The literature has explored various concepts related to WRQoL, including psychological flexibility^2^, well-being^1^, work engagement^16^, job stress^17^, work-family conflict^18^, and job satisfaction^3^.

Perceived social support can be considered an important factor in determining WRQoL. Perceived social support is an individualized assessment reflecting the degree to which an individual considers that others, such as family and friends, provide support or care for them^19^. Social support signifies the presence or accessibility of individuals around whom we can count, and who communicate their concern, value, and affection^20^. Social support serves as a bridge between work and social life^21^. According to the Conservation of Resources (COR) theory, social support can have an impact on outcomes due to its significance as a vital and recognized personal resource^22^. In addition, the perception of social support entails cognitive processes. For example, following a negative occurrence, an individual may evaluate their social support as insufficient, thus leading to feelings of depression. It is a significant resource that mitigates the adverse impacts of stressful experiences, including those related to work life^23^. An employee confronted with a challenging work circumstance may maintain composure and demonstrate a problem-solving mindset owing to the support^24^. Numerous research exist in the literature regarding sources of support. In one study, management and supervisory support significantly contributed to managing work demands and family responsibilities^25^. A separate study examined perceived workplace support, perceived family support, and perceived supervisory instrumental support, all of which significantly influenced work-life balance. Another study demonstrated that personal social support, managerial support, and colleague support considerably and positively influence work-life balance^12^. Nonetheless, sources of support extend beyond the workplace. Support from family, friends, and significant others can also influence work life. These resources affect individuals’ life satisfaction ^11^.

There is a lack of research in the existing literature that examines the impact of perceived social support on WRQoL. Nevertheless, the research has explored the relationships between perceived social support and several variables related to satisfaction and balance in both work and personal life. The literature indicates a significant correlation between perceived social support and work-life balance^12^, quality of life^26^, and life satisfaction^27^. According to the social support approach, individuals who experience greater amounts of social support from their social network generally exhibit enhanced psychological well-being, decreased stress, and improved coping strategies^28^. According to the COR theory, psychological distress can be considered as an important factor that can impact individuals’ everyday work experiences, leads to resource depletion, and adversely influence their overall quality of life^13,29^. Within the framework of the working setting, this would result in an enhanced quality of life in the workplace. When employees perceive a sense of support from their families, friends, and significant others outside of the workplace, they could be more inclined to experience satisfaction. A recent study found a negative correlation between perceived social support and psychological distress^27^. Increased perceptions of social support could assist individuals in enduring stressful circumstances and mitigate adverse feelings^30^, hence fostering improved social interactions in work environments like educational institutions and sustaining current social connections. The following hypotheses were established based on the literature.

### H1

Perceived social support has a significant positive effect on work-related quality of life.

### H2

Perceived social support has a significant negative effect on psychological distress.

Psychological distress can have a vital role in determining WRQoL and its indirect effect on the effect of perceived social support on WRQoL. It refers to a broad range of emotional suffering that is marked by depressed attitudes, such as feelings of melancholy and hopelessness, as well as signs of anxiety, such as tension^31^. It encompasses the consequences of an individual’s psychobiological, cognitive, and social interactions. It is frequently linked to inaccuracies in the processing of information, such as attention deficit and cognitive bias^32^. Accordingly, a significant degree of psychological distress can have a negative effect on employees’ work performance, ultimately affecting their overall WRQoL. Existing research has predominantly focused on investigating psychological distress within the realm of health, as evidenced by studies conducted^33,34^. There appears to be a lack of comprehensive studies across different sectors. Additionally, some studies have been carried out including employees. For instance, psychological distress has been studied concerning job performance, psychological resilience, employee creativity^35^, and turnover intention^36^. However, it is imperative to examine the concept of psychological distress specifically within the education sector, which is distinct from other sectors. Since the onset of the COVID-19 pandemic, teachers have encountered a range of physical and psychological challenges, including heightened stress, exhaustion, anxiety, depression, feelings of hopelessness, and disrupted sleep patterns^37,38^. Nevertheless, it appears that the research conducted in the field of education is constrained.

The Job Demands-Resources (JD-R) theory posits that work-related well-being and job performance are influenced by two primary categories: job demands and job resources. Psychological distress might be regarded because of increased job demands and insufficient job resources^39^. Increased job demands, such as an intense workload or emotional demands, might result in higher psychological distress. In the same manner, insufficient resources, such as a lack of social support, can also play a role in causing psychological distress. Psychological distress could have a negative impact on WRQoL by decreasing job satisfaction and bringing job-related stress. Additionally, The COR suggests that individuals have a natural inclination to protect, maintain, and gather resources. It further states that the loss of resources causes stress, while the acquisition of resources promotes well-being. Perceived social support is a significant personal resource that individuals want to maintain and protect within the framework of social support^13^. For instance, as individuals experience greater perceived social support, they are more likely to acquire a significant personal resource. This resource can serve as a buffer against the depletion of other resources and can contribute to enhanced psychological well-being. But, if individuals perceive a lack of social support, they may also perceive a loss of this important resource, which can result in increased psychological distress. Psychological distress can have a negative impact on WRQoL. The following hypotheses were established based on the literature and JD-R and COR theory.

### H3

Psychological distress has a negative effect on work-related quality of life.

### H4

Psychological distress plays a mediating role in the relationship between perceived social support and work-related quality of life.

Previous research indicates that gender differences have a notable influence on how perceived social support is experienced, and how it subsequently affects psychological well-being. Research has shown that females frequently report greater levels of perceived social support in comparison to males, which can impact their experience of psychological distress^14^. On the other hand, a study found no significant correlation between perceived social support and psychological distress in males, but there was a significant correlation between the variables in females^40^. In times of stress, females frequently demand support more than males, which might have varying effects on WRQoL. Moreover, drawing upon the JD-R theory and the Spillover theory, it may be hypothesized that the impact of perceived social support on WRQoL could differ depending on gender^4,39^^.^ Based on the JD-R theory, social support is regarded as a job resource that can mitigate the adverse effects of job demands on employee well-being^39^. The social support that comes from family, friends, and significant other can be considered as a personal resource that might affect work performance in the JD-R Theory. Additionally, the spillover theory posits that experiences in one area of life, such as work, have the potential to impact experiences in other areas of life, such as overall quality of life^4^. A study indicated that females tend to report higher amounts of social support compared to males^14^. Another study demonstrated that females (ß = 0.49, p < 0.001) with higher social support are more likely to experience higher life satisfaction compared to males (ß = 0.26, p < 0.05)^11^. Accordingly, the utilization of social support and the impact of work experiences on quality of life may vary based on gender, and these differences can affect the relationship between perceived social support and WRQoL. The following hypotheses were formed based on the literature, JD-R theory and spillover theory:

### H5

The relationship between perceived social support and psychological distress is stronger in females compared to males.

### H6

The relationship between psychological distress and work-related quality of life is stronger in females compared to males.

### H7

The relationship between perceived social support and work-related quality of life is stronger in females compared to males.

## Materials and methods

### Instruments

The study utilizes three scales to measure the variables. The scale from Van Laar et al.^41^ has been employed for the measurement of WRQoL, Kessler et al.^42^ for the measurement of psychological distress, and Zimet et al.^19^ for the measurement of perceived social support. Previous Turkish translations have been applied for the assessment of WRQoL^43^, Multidimensional Perceived Social Support^44^, and psychological distress^45^.

*Work-related quality of life:* The scale comprises 23 items. It consists of 6 subscales, including Job and Career Satisfaction, General Well-Being, Home–Work Interface, Stress at Work, Control at Work, and Working Conditions. Job and Career Satisfaction includes 6 items. Some of the items in this subscale are as follows: *“I am satisfied with the career opportunities available to me at the organization, I have the opportunity to use my abilities at work”*. General Well-Being includes 6 items. The following are examples of the items: *“Generally, things work out well for me, I am satisfied with my life”.* Home–Work Interface includes 3 items. The following are some of the items: *“My employer provides adequate facilities and flexibility for me to fit work in around my family life, My current working hours/patterns suit my personal circumstances”.* Stress at Work contains 2 items. The items are as follows: *“I often feel under pressure at work, I often feel excessive levels of stress at work”.* Control at Work contains 3 items. Some items are as follows: *“I am involved in decisions that affect me in my own area of work, I am involved in decisions that affect members of the public in my own area of work”.* Working Conditions contains 3 items. These are some items for working conditions: *“The working conditions are satisfactory, I work in a safe environment”.* Overall Cronbach Alpha’s value is 0.91. The Cronbach Alpha coefficients of the subscales range from 0.76 to 0.91. A fully anchored five-point Likert-type rating scale was applied to measure WRQoL (1 = Strongly disagree, 5 = Strongly agree).

*Multidimensional perceived social support:* The scale comprises 12 items. The scale comprises three subscales, each relating to a distinct source of support: family, friends, and significant others. The family subscale includes 4 items. Some items are as follows: *“My family really tries to help me, I get the emotional help and support I need from my family”*. The friends’ subscale includes 4 items. These are some items: *“My friends really try to help me I can count on my friends when things go wrong”*. The significant other subscale contains 4 items. The following are some of the items: *“There is a special person who is around when I am in need, There is a special person with whom I can share my joys and sorrows”.* Overall Cronbach Alpha’s value is 0.88. The Cronbach Alpha values for the subscales range from 0.85 to 0.91. The assessment for multidimensional perceived social support was given using a 5-point Likert-type scale, with choices ranging from strongly disagree (1) to strongly agree (7).

*Psychological distress:* The scale is single-dimensional and has 10 items. Some items are as follows: *“About how often did you feel tired out for no good reason? About how often did you feel nervous? About how often did you feel so nervous that nothing could calm you down? About how often did you feel hopeless?”*. Overall Cronbach Alpha’s value is 0.92. The evaluation of psychological distress was conducted using a 5-point Likert-type scale that ranged from never (1) to always (5).

### Research model

**Fig. 1:**
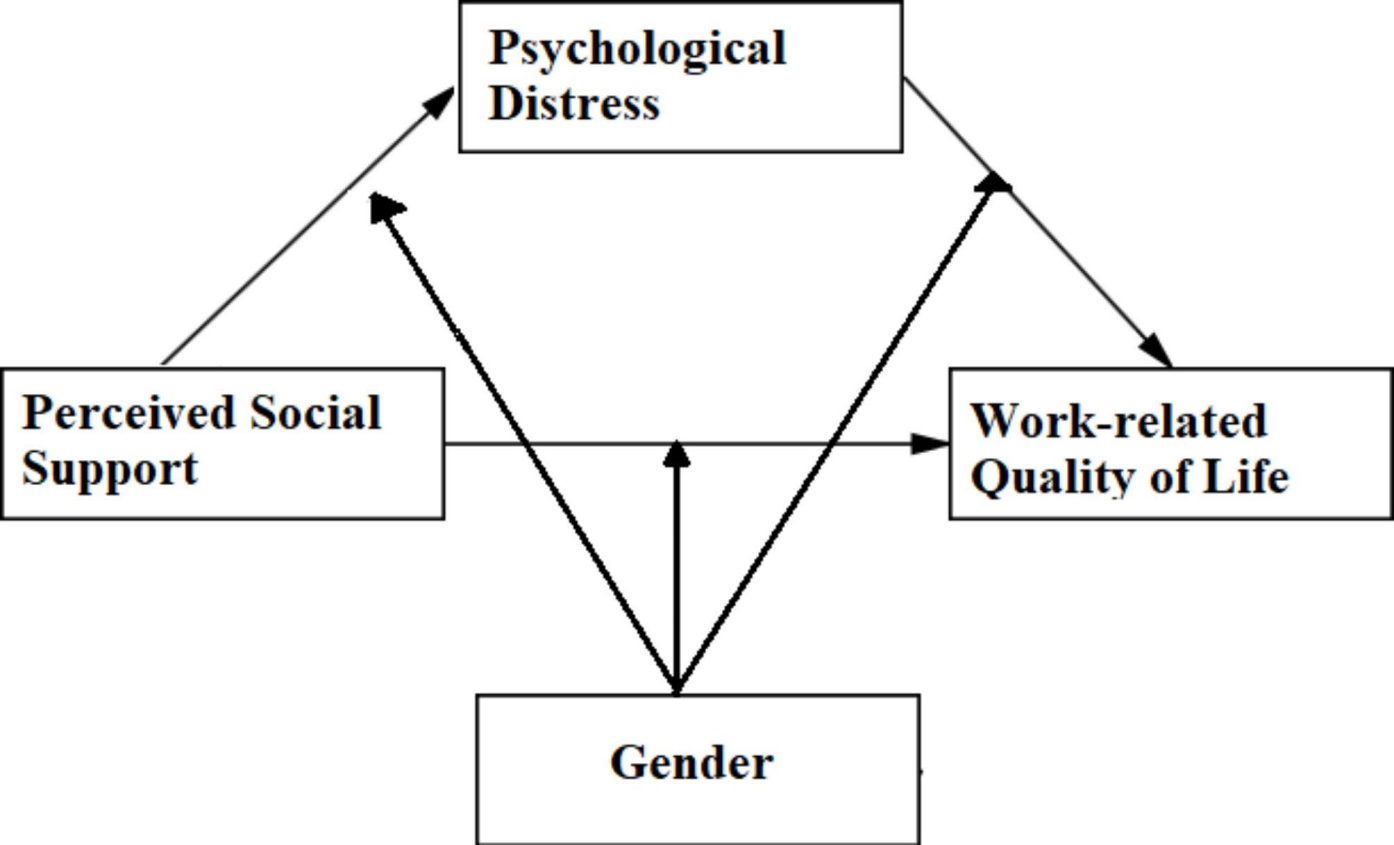
Research model.

The study’s primary focus is on the relationship between perceived social support, psychological distress, and WRQoL, as elucidated in the conceptual framework. Based on the existing literature, Fig. 1 illustrates the research model. In the model, perceived social support was defined as an explanatory variable, psychological distress was considered as both an explanatory and dependent variable, and WRQoL was regarded as the dependent variable. Moreover, it is shown that these relationships varied by gender, suggesting that the strength or direction of the relationships between variables may vary for male and female individuals.

### Sampling and data collection

The study employed the survey approach, which is a quantitative data collection method, to evaluate the model. It applied a cross-sectional approach, gathering data from the teachers at a singular period. The data was acquired by utilization of the convenience sampling technique. When identifying the population for the study, it was preferable to concentrate on a certain occupational group, as each profession can have distinct impacts on individuals. The study’s population comprised teachers located in Ordu, Türkiye.

A pilot study was carried out to verify the reliability and content validity of the instruments before formally gathering the data. The pilot research was performed using a different sample from the main research sample. The pilot study sample comprises 60 high school teachers assigned by the Ministry of National Education in Ordu, Türkiye. The teachers’ departments are varied. Data was gathered through visits to local schools. The main objective was to evaluate the clarity of the items. The reliability of the scales was assessed by Cronbach’s alpha values, which exceeded 0.70. The feedback from the pilot research and expert evaluations were assessed for content validity. The clarity and suitability of the items were examined. Participant feedback enabled the clarification of items, while expert assessments ensured the content validity. Cronbach’s alpha was computed to assess internal consistency, while expert evaluations and feedback verified content validity. Accordingly, the pilot study verified the minimal presence of discrepancies and established reliability and content validity. After the pilot study, the data collection phase started. Data collection involved conducting school visits to teachers employed in Ordu and distributing survey forms. Twelve high schools were visited for data collection. In the end, a total of 420 surveys were acquired from teachers. Prior to doing the study, the acquired data was assessed using data-cleaning techniques. The analysis focused on evaluating standard deviation, extreme values, outliers, skewness, and kurtosis data within this particular context. Consequently, the study’s sample comprised 401 teachers. The study was conducted in accordance with the principles outlined in the Declaration of Helsinki and got ethics approval from the Ethics Committee of Ordu University (23.02.2024–33/2024). Also, informed consent was obtained from all participants.

### Common method variance

The survey was designed considering the recommendation of Podsakoff, MacKenzie, and Podsakoff (2012) to reduce the occurrence of common method variance^46^^.^ For instance, the wording of survey questions might influence participants’ comprehension and responses. Therefore, precautions were implemented regarding the wording of items. Positive and negative items have been distributed fairly. Precautions were implemented to prevent the focus of responses from being in a singular direction. Also, efforts were made to ensure that the scale items had no presence of ambiguity. Additionally, anonymity was implemented to mitigate participants’ evaluation anxiety and to minimize social desirability bias. Plus, proximal separation was prioritized. The independent and dependent variables were located on separate pages. Measures were implemented to prevent participants from forming a close association between the questions and inferring a cause-and-effect relationship between the research variables. Consequently, several procedures have been undertaken to get more consistent and reliable responses. Furthermore, Harman’s one-factor test was utilized to detect possible common method bias. Firstly, the scale components were merged into a single factor. Afterward, the measurement model of the resulting single factor was examined in relation to the existence of common method bias. The results showed a significantly inadequate model (χ2 = 5342.930, df = 557; (χ2)/df = 9.592; root mean square error of approximation (RMSEA) = 0.147; comparative fit index (CFI) = 0.473; tucker lewis index (TLI) = 0.437). The result suggests that the existence of common method bias is not a substantial concern in the sample for this study.

### Control variables

Previous studies on WRQoL have examined the impact of demographic factors such as age and work experience^47^. The decision to include age and work experience as control factors in this analysis was based on previous research and their potential to create confounding effects. The factors have been diligently monitored during each analysis. The control variables were regarded as covariates, and their direct impacts on the dependent variables were defined. This provided that correlations between the independent and dependent variables remained unaffected by these factors, facilitating a more explicit interpretation of the findings.

## Results

### Data analysis

Firstly, demographic data regarding the sample is provided. The study sample has a total of 401 participants. Males are designated as 1 and females as 2 in gender coding. Of the participants, 182 (45.4%) were male and 219 (54.6%) were female. The results indicate a balanced gender distribution throughout the sample. The study sample is categorized by age groups as follows: There were 44 participants (11%) aged 18–24, 130 participants (32.4%) aged 25–35, 130 participants (32.4%) aged 36–45, 84 participants (20.9%) aged 46–55, and 13 people (3.2%) aged 56 and over. This distribution indicates that the sample encompasses a broad age spectrum. The allocation of teachers in the research sample based on their work experience is as follows: 121 teachers (30.2%) have 0–5 years of experience, 70 teachers (17.5%) have 6–10 years of experience, and a subset has 11–15 years of experience. 50 teachers (12.5%), 53 teachers (13.2%) with 16–20 years of experience, 47 teachers (11.7%) with 21–25 years of experience, and 60 teachers (15%) with 26 years of experience or more. This distribution indicates that the sample encompasses diverse levels of professional experience.

Furthermore, the hypotheses were evaluated Structural Equation Model in AMOS version 23, employing the maximum likelihood estimate method. The path analysis was conducted using bootstrap standard errors with 5000 resamplings. The method of bootstrapping has been suggested as the preferred approach for testing mediation, as proposed by MacKinnon et al.^48^.

### Validity and reliability

The analysis was conducted using AMOS version 23, using the following indicators: (a) Chi-square divided by degrees of freedom (χ2/df) (b) CFI (c) TLI (d) RMSEA. Upon conducting the initial assessment of the measurement model, it was found that several items displayed factor loadings below 0.55. Comrey and Lee (1992) assert that items with factor loadings exceeding 0.55 are considered to have significant factor loadings, showing that these items are strongly indicative of the underlying construct^49^. This means the items exhibit a robust connection with the underlying construct, signifying that it serves as a reliable indication of the construct being assessed. Therefore, 10 items which are WRQL1, WRQL3, WRQL6, WRQL7, WRQL8, WRQL9, WRQL19, WRQL20 (respectively, *I have a clear set of goals and aims to enable me to do my job, I have the opportunity to use my abilities at work, My current working hours/patterns suit my personal circumstances, I often feel under pressure at work, When I have done a good job it is acknowledged by my line manager, Recently, I have been feeling unhappy and depressed, I often feel excessive levels of stress at work, I am satisfied with the training I receive in order to perform my present job*) were removed from the measurement model. Furthermore, after examining the regression weights for PD3 (*About how often did you feel so nervous that nothing could calm you down?*), it becomes apparent that it leads to cross-loading which is a situation in which an item exhibits a significant factor loading on multiple factors, resulting in ambiguity regarding its association with a specific component. Consequently, PD3 was eliminated from the model. While examining the factor loadings and modification indices of the measurement model, the definitive items were identified. The measurement model exhibited exceptional fit according to multiple indices (χ2/df = 2.247; CFI = 0.925; TLI = 0.918; RMSEA = 0.056)^50^. The study’s findings provided solid evidence supporting the 35-factor structure of the measurement model.

Table 1 displays the mean, standard deviation, and correlations among the study variables, as well as the validity and reliability analysis results of the study. A significant correlation was seen between psychological distress and age (r = − 0.195; p < 0.01), work experience (r = − 0.186; p < 0.01), and perceived social support (r = − 0.304; p < 0.01). Furthermore, another significant correlation was observed between WRQoL and perceived social support (r = 0.395; p < 0.01), as well as psychological distress (r = − 0.372; p < 0.01).

**Table 1:** Descriptive statistics.

		Mean	SD	AVE	CR	Skewness	Kurtosis	Cronbach Alpha (α)	1	2	3	4	5	6
1	Gender	1.55	0.498						1					
2	Age	2.73	1.016						0.306**	1				
3	WE	3.04	1.826						0.299**	0.873**	1			
4	PSS	5.643	1.140	0.710	0.964	− 0.724	− 0.158	0.904	− 0.07	− 0.028	0.003	1		
5	PD	2.034	0.648	0.515	0.904	0.438	− 0.542	0.904	− 0.059	− 0.195**	− 0.186**	− 0.304**	1	
6	WRQoL	3.561	0.685	0.556	0.949	− 0.086	− 0.401	0.922	0.016	− 0.002	0.014	0.395**	− 0.372**	1

**. Correlation is significant at the 0.01 level (2-tailed) PD = Psychological Distress, PSS = Perceived Social Support, WRQoL = Work-related quality of life, WE = Work experience.

For the purposes of evaluating convergent validity, the study assessed the average variances extracted (AVE) for each construct, as recommended by Fornell and Larcker in 1981. AVE values were found to be greater than 0.5, ranging from 0.515 to 0.710^51^. Composite reliability evaluates the degree of internal consistency among the items that assess a specific construct^52^. In order to assess composite reliability, the construct reliability (CR) is measured for each construct, as described by Straub et al.^53^. The CR values above 0.70, range from 0.904 to 0.964. Furthermore, the Cronbach Alpha values have been evaluated to determine the reliability of the scales. The values of Cronbach’s alpha and CR surpass the threshold of 0.70, therefore satisfying the internal consistency requirements proposed by Nunnally^54^. The findings confirm that items are acceptable, and the structures are both reliable and consistent. Discriminant validity is confirmed when the square root of the AVE values of a scale is greater than the correlations observed between the scales. Upon examining the discriminant validity scores of the variables, it emerged that the values surpassed the correlation values. The results of this study demonstrate that discriminant validity has been verified^52^.

Along with this, a study was done to analyze the variance inflation factors (VIF) to identify the existence of multicollinearity. It is imperative to assess the VIF of each construct. The VIF values of the independent factors that affected the dependent variable of WRQoL were determined to be less than 10 (Perceived social support = 1.102 and psychological distress = 1.102). According to Neter, Wasserman, and Kutner (1989), the lack of a multicollinearity problem seems to be attributed to the values being less than 10^55^.

### Structural equation model

The measurement model’s satisfactory conformity instills confidence in proceeding with evaluating the study hypotheses using SEM. The current study examined the direct and indirect correlations between the variables. In order to test the proposed hypotheses, a path analysis was performed using AMOS version 23. The fit indices demonstrate that the model fits the data sufficiently, as indicated by the following values: χ2/df = 2.247, TLI = 0.918, CFI = 0.925, RMSEA = 0.056^50^.The structural model indicated that perceived social support had a statistically significant impact on WRQoL (β = 0.271, p < 0.001) with a 95% confidence interval of [0.243, 0.502] and psychological distress (β = − 0.317, p < 0.001) with a 95% confidence interval of [− 0.513, − 0.292]. Hence, H_1_ and H_2_ were supported. The effect of psychological distress on WRQoL was statistically significant (β = − 0.213, p < 0.010), with a 95% confidence interval of [− 0.301, − 0.063]. This result revealed that H_3_ was supported. The overall structural model explained 16.7% of the variance in WRQoL. The results of the path analysis are displayed in Table 2.

**Table 2:** Path analysis results.

Path	Direct effect	S.E	C.R	p
PSS → WLQ	0.271	0.062	4.383	0.000
PSS → PD	− 0.317	0.055	− 5.783	0.000
PD → WLQ	− 0.213	0.070	− 3.056	0.002

Moreover, an analysis of the indirect impact has been performed by the utilization of the SEM bootstrapping method. A mediating effect analysis was carried out to determine the involvement of psychological distress in the impact of perceived social support on WRQoL (H_4_). Table 3 shows the standardized indirect effect values. The preliminary evaluation of the indirect effect resulted in a coefficient value of ß = 0.074 for PSS → PD → WLQ. Following that, 5000 re-samples were taken to assess the lower and upper bounds with a confidence level of 95% to test the significance of the indirect effect. Therefore, it was concluded that the indirect effect was statistically significant, as evidenced by the lack of the value "0" in the PSS → PD → WLQ (ß = 0.074; LLCI: 0.025; ULCI: 0.130) path. This finding supported H_4_.

**Table 3:** Bootstrap Results.

Path	Standardized Indirect effect	BC Confidence Intervals (95%)	
		Lower Bound	Upper Bound
PSS → PD → WLQ	0.074	0.025	0.130

### Measurement invariance

Measurement invariance was evaluated by multiple-group analysis using AMOS to determine if the study constructs had consistent interpretations across all respondent groups, sorted by gender. Measurement invariance can be evaluated by examining the statistical significance of the change in χ2 between two nested models. Nevertheless, the χ2 test is very responsive to minor, inconsequential deviations from an ideal model in substantial samples^56^. Hence, employing CFI difference tests is recommended instead of χ2 and verify that the resultant difference values are less than 0.01 or equal^57^. Accordingly, the study assessed measurement invariance by computing the difference in ΔCFI between the restricted and unconstrained models. The study examined configural, metric, and scalar invariances. Configural invariance assesses if a distinct group of participants produces the same configuration of factors^58^. Configural invariance was checked through model fit indices. The values surpassed the model fit indices’ thresholds as shown in Table 4^59^. This suggests that the measurement model successfully demonstrated configural invariance across all groups. Metric invariance assumes that the factor loading is the same across different groups^50^. Metric invariance was assessed by imposing constraints on factor loadings in the measurement model. Next, the differences in ΔCFI between the restricted and unconstrained models are computed. ΔCFI value is less than 0.01, it can be concluded that metric invariance can be claimed^57^. The scalar test has been conducted after completing the configural and metric tests. Scalar invariance postulates that the measurement intercepts are identical across different groups. The findings displayed in Table 4 indicate that there is configural, metric, and scalar invariance among participants from different gender groups, as determined by the ΔCFI criteria (ΔCFI ≤ 0.01).

**Table 4:** Measurement invariance results.

Moderator Variable	Model	χ2	df	χ2/df	TLI	RMSEA	CFI	ΔCFI
Gender	Unconstrained	1713.197	1042	1.644	0.918	0.040	0.928	
	Configural	1756.492	1068	1.645	0.918	0.040	0.926	0.002
	Metric	1789.040	1103	1.622	0.921	0.039	0.926	0.000
	Scalar	1882.265	1141	1.650	0.917	0.040	0.920	0.006

### Multigroup analysis

A multigroup analysis was conducted to evaluate the impact of gender. Two nested models were constructed for each group, using the approaches recommended by Alrawad et al.^60^. A model was constructed with the imposition of equal restrictions on the regression weights, while the second model was constructed without any constraints. Simultaneously, constraints were applied to all regression weights to examine interaction effects for the model. Subsequently, Δχ2 was computed. Also, separate tests were conducted on the PSS → WLQ, PSS → PD, and PD → WLQ paths to determine the presence of an interaction effect. The constrained and unconstrained models were similar for PSS → PD (Δχ2 = 1.038, df = 1, p = 0.308) and PSS → WLQ (Δχ2 = 0.466, df = 1, p = 0.495). The relationship between psychological distress and WRQoL differs based on gender (Δχ2 = 7.786, df = 1, p < 0.01). The impact of psychological distress on WRQoL is higher for females. Thus, hypothesis results are shown in Table 5.

**Table 5:** Chi-square difference test for multi-group analysis.

Hypotheses	Paths	β (male)	p	β (female)	p	Δχ2	Δ df	Result
	Model 1				0.040	8.298	3	
H_5_	PSS → PD	− 0.383	0.000	− 0.271	0.000	1.038	1	Rejected
H_6_	PD → WLQ	− 0.009	0.917	− 0.386	0.000	7.786	1	Supported
H_7_	PSS → WLQ	0.325	0.000	0.239	0.004	0.466	1	Rejected

### Control variables

Regarding the control variables, the analysis revealed that there was no significant correlation between participants’ age (β = − 0.055, p = 0.384) and work experience (β = 0.001, p = 0.975) with WRQoL. The results of all the hypotheses remained similar, even after considering age and work experience as control variables. The absence of confounding effects is demonstrated by the absence of substantial changes in the estimates when considering age and work experience.

## Discussion

This study presents significant data about the relationships between work and social life, investigating the relationship between WRQoL, perceived social support, and psychological distress. The findings indicate that perceived social support positively influences WRQoL, with psychological distress serving as a mediator factor in this relationship. The results supported that perceived social support has a significant impact on both WRQoL (H_1_) and psychological distress (H_2_). The benefits of perceived social support in the workplace are most visible when derived from family, friends, and significant others. The study offers a novel viewpoint on the JD-R theory by assessing perceived social support as both a job resource and a factor that enhances individuals’ coping mechanisms in response to job demands. This study examines the impact of social support from family, friends, and significant others on WRQoL, highlighting that social support is an essential determinant of work-life quality within the JD-R model^39^. The finding that individuals experiencing social support exhibit less psychological distress suggests that supportive interactions enhance resilience to stress. This study, in addition to Hobfoll’s theory on resource conservation tendency, investigates the positive effects of social support on WRQoL, illustrating that social support functions not only to preserve current resources but also as a catalyst for resource expansion, thereby strengthening employees’ resilience and alleviating psychological distress in the workplace^13^. The findings are in parallel with previous research that has shown that social support acts as a buffer against psychological distress^27,28^. The findings indicated a notable negative correlation between psychological distress and WRQoL (H_3_). An individual’s overall stress adversely impacts their work life, diminishing their experience and satisfaction at work. When individuals struggle to fully detach from non-work pressures, these challenges adversely affect motivation, productivity, and job satisfaction^4^. It is also consistent with COR theory. It posits that individuals endeavor to preserve their resources and encounter stress when these resources are diminished or endangered. The decline in work-life quality associated with heightened psychological distress indicates that a shortage of resources adversely impacts individual satisfaction, stress management, and overall quality of life. This indicates that inadequate present resources adversely impact individuals’ quality of work life^13,29^. The psychological challenges encountered by individuals in different sectors, like education, and the post-COVID-19 epidemic align with the findings of this study^37,38^. The anxiety and stress encountered by teachers substantially affect WRQoL. Consequently, enhancing psychological support services in workplaces and increasing employees’ social support resources is essential for improving the WRQoL. According to COR and the JD-R theory, the results supported the hypothesis that psychological distress acts as a mediator in the relationship between perceived social support and WRQoL (H_4_). The COR theory posits that social support enhances WRQoL by preserving individuals’ existing resources and alleviating psychological distress, whereas the JD-R model contends that social support functions as a resource essential for individuals to fulfill job demands. The study’s findings indicate that social support serves as a resource and indirectly enhances work-life quality by alleviating individuals’ psychological stress. Supportive relationships mitigate the impact of stress and enhance employees’ sense of strength and competence, so elevating their level of satisfaction^13,39^. Unfortunately, the study unveiled that the relationship between perceived social support and psychological distress does not vary by gender (H_5_). The complex relationship of socio-cultural, biological, and organizational factors may determine the perception and utilization of social support. The results demonstrate that the relationship between psychological distress and WRQoL varies by gender (H_6_). More precisely, the study revealed that the influence of psychological distress on females’ WRQoL was greater. This is consistent with prior research that suggests there are variations by gender in how psychological distress is experienced. Females generally exhibit higher levels of psychological distress in comparison to males, which can manifest in diverse impacts on their well-being and quality of life^11,40^. The findings did not support the fact that the impact of perceived social support on WRQoL might differ based on gender (H_7_). The supportive components of relationships may have a constant impact on both men and female regarding their WRQoL. Additionally, unexplored variables such as individual personality traits, coping mechanisms, or organizational culture may have had a substantial impact on the relationship between perceived social support and WRQoL, potentially overshadowing any gender differences.

The findings demonstrated that neither age nor work experience had a significant relationship with WRQoL. This indicates that these control factors did not significantly impact the interpretation of the primary effects and hypotheses in the model. The lack of substantial correlations between these control variables and WRQoL suggests that other factors may be more influential in shaping WRQoL, irrespective of demographic variables like age and work experience. The inclusion of age and work experience as control factors ensures a comprehensive model and their absence would not change the principal findings.

### Theoretical implications

This study filled a gap in the existing knowledge by investigating the mechanism by which perceived social support affects WRQoL, and the role of psychological distress as a mediator. The findings contributed to the theoretical understanding of the psychological mechanisms that link social support to work-related well-being outcomes. It evaluated different sources of support on WRQoL. The study also contributed to the literature by examining potential gender differences among the variables. Furthermore, the study expanded upon the JD-R theory by investigating the mediator role of psychological distress in the relationship between perceived social support and WRQoL. Moreover, the study emphasized the spillover effects of psychological distress from work to personal life and its influence on WRQoL.

### Managerial implications

The study highlights the significance of organizational strategies and interventions that aim to promote perceived social support, manage psychological distress, and consider gender variations to enhance WRQoL. The findings reveal that psychological distress adversely impacts the WRQoL of female employees. Therefore, efforts that minimize this gender disparity are essential. Stress management programs and psychological support services could decrease the adverse impacts of distress, especially among female employees. Furthermore, remedies that enhance perceived social support can improve WRQoL, as evidenced by the identified correlation between social support and reduced psychological distress in the study. Organizations can gain advantages by conducting training and development programs focused on improving social support abilities among managers and employees. Organizations can formulate policies that foster an optimal balance between work and personal life, while simultaneously diminishing stress caused by work. Flexible work arrangements, such as remote work options or flexible hours, along with supportive leadership and effective communication, can enhance WRQoL.

### Limitations and scope for future research

Although this study offers appealing insights, it is important to understand its limitations. The study’s cross-sectional design restricts the capacity to establish causal relationships. Subsequent investigations utilizing longitudinal designs could offer stronger evidence of the relationship among perceived social support, psychological distress, and WRQoL. Furthermore, the study specifically targeted the education sector, and its findings may not be applicable to other professional sectors. Further research should explore these relationships across different industries. This study is limited by the identification of family, friends, and significant others as sources of social support. Additional sources were excluded from the investigation. Lastly, it would be beneficial to examine the moderating roles of individual and organizational characteristics, including personality traits, coping mechanisms, and leadership styles.

## Conclusion

The study investigated the impact of perceived social support on WRQoL, mediating the role of psychological distress in this relationship, while also examining potential gender differences. The study is crucial in enhancing our comprehension of the fundamental mechanisms and gender-specific dynamics that impact WRQoL. The study’s results provided evidence for the direct impact of the variables and supported that psychological distress acts as a mediator in the relationship between perceived social support and WRQoL. Moreover, the study unveiled that the effect of psychological distress on WRQoL was stronger among females, underscoring the significance of considering gender differences. From a theoretical perspective, the study also enriched the existing frameworks by integrating COR, JD-R theory, and spillover theory. Fundamentally, the study yielded significant insights into decision-makers and policymakers. The findings showed the necessity of establishing favorable work settings that promote social support and psychological well-being. Moreover, the results emphasized the significance of creating interventions and support systems tailored to each gender to tackle the unique requirements and difficulties encountered by male and female employees in the workplace.

## Data Availability

The datasets produced and examined in the present study can be obtained from the corresponding author upon a reasonable request.
